# Impact of bisphosphonate drugs on dental implant healing and peri-implant hard and soft tissues: a systematic review

**DOI:** 10.1186/s12903-022-02330-y

**Published:** 2022-07-17

**Authors:** Luca Fiorillo, Marco Cicciù, Tolga Fikret Tözüm, Cesare D’Amico, Giacomo Oteri, Gabriele Cervino

**Affiliations:** 1 Department of Biomedical and Dental Sciences, Morphological and Functional Images, University of Messina, Via Consolare Valeria, 1, 98100 Messina, Italy; 2 Multidisciplinary Department of Medical-Surgical and Odontostomatological Specialties, University of Campania “Luigi Vanvitelli”, 80121 Naples, Italy; 3 Department of Periodontics, University of Illinois at Chicago, College of Dentistry, Chicago, IL USA

**Keywords:** Dental implant, Bisphosphonate, Osteoporosis, Osseointegration, Oral surgery, Bone tissue

## Abstract

**Objective:**

Implantology represents the gold standard for oral rehabilitation, unfortunately, often, despite there are no local contraindications to this type of rehabilitation, there are uncertainties regarding the general health of our patients. Many patients nowadays take bisphosphonate drugs, often without first seeking advice from an oral surgeon or a dentist. The purpose of this review is precisely to highlight any contraindications to this type of treatment reported in the literature, in patients who take or have taken bisphosphonate drugs.

**Methods:**

For this study the scientific information sources were consulted using as search terms “(“bisphosphonate AND “dental implant”)”, obtaining 312 results, these were subsequently skimmed according to the inclusion and exclusion criteria, and further evaluated their relevance to the study and the presence of requested outcomes.

**Results:**

Only 9 manuscripts (RCTs, Multicentric studies and Clinical Trials) were included in this review, as they respected the parameters of this review, they were analyzed and it was possible to draw important results from them. Surely from this study it is understood that the use of bisphosphonate drugs does not represent an absolute contraindication to implant therapy, it is evident how adequate pharmacological prophylaxis, and an adequate protocol reduce the risks regarding implant failures. Furthermore, the values of marginal bone loss over time seem, even if not statistically significant, to be better in implant rehabilitation with bisphosphonate drugs association. Only a few molecules like risedronate, or corticosteroids, or some conditions like smoking or diabetes have shown a high risk of surgical failure.

**Conclusion:**

Although this study considered different studies for a total of 378 patients and at least 1687 different dental implants, showing better results in some cases for dental implant therapy in cases of bisphosphonate intake, further clinical, randomized and multicentric studies are needed, with longer follow-ups, to fully clarify this situation which often negatively affects the quality of life of our patients and places clinicians in the face of doubts.

## Introduction

### Rationale

Implantology is a rehabilitation procedure aimed at those who have lost their natural teeth through the use of titanium fixture. Artificial teeth are designed to replace real teeth that are missing both in terms of aesthetics and chewing function. State-of-the-art dental implantology techniques, such as those practiced in dental clinics, allow permanent rehabilitation of chewing and avoid the hassles associated with the use of removable prostheses, ensuring an aesthetic result equal to that of natural teeth [1, 2].

In very general, dental implant surgery consists of placement of a titanium dental implant into the bone of the affected dental arch with the aim of bone integration (osseointegrated implantology).Usually, the implant integration time varies according to the affected bone: maxilla (4–6 months); mandibular (3–4 months) [3, 4].

There are several factors effecting the estimated healing time by the implantologist, that can slow down osseointegration [5]. Complete osseointegration of dental implants is successful in the vast majority of cases. International studies based on the scientific and rigorous evaluation of over 1,200,000 implants have indicated that the average success rate is over 98%. For smokers, the success rate drops to 85% [6]. The implantology intervention is one of the practices related to oral surgery, and represents a surgical intervention on the hard and soft tissues of the maxillary bones [7, 8].

Systemic or general contraindications, on the other hand, have to do with the patient's systemic health and any pharmacological or radiation therapies. Among these, one that jumps to attention and that is often underestimated during a first dental visit is the current or past intake (considering their half-life) of bisphosphonate drugs. Bisphosphonates are a category of drugs that inhibit bone remodeling, which is why they are used for the treatment of various metabolic and oncological disorders, affecting bone tissue; osteoporosis, osteopenia, osteogenesis imperfecta, Paget's disease, multiple myeloma, malignant hypercalcemia and bone metastases following cancer [9, 10].

Bisphosphonates are very effective drugs that reduce the incidence of fractures, as they increase bone density. They have the characteristic of binding to calcium and for this reason they are deposited in the bone tissue. Bisphosphonates can cause bone lesions of the maxilla and/or jaw associated with local signs and symptoms of various types and severities, such as ulceration of the oral mucosa that lines the bone, exposure of bone in the oral cavity, pain in the teeth and/or to the jaw/jaw bones, swelling or inflammation, numbness or a feeling of 'heavy jaw', increased tooth mobility, tooth loss [11]. Before undertaking any surgical-implant therapy, it is necessary to acquire and analyze a complete medical history of the patient and, in the case of the presence of bisphosphonate therapy, the duration of treatment must be confirmed, as well as the route of administration of the same.

### Objectives

The purpose of this systematic review is to analyze literature to understand the impact of Bisphosphonate drugs on dental implant therapy, with the aim to evaluate different clinical and radiographic outcomes reported in literature. The type of studies that were taken under consideration included: studies with test and control groups, Randomized Controlled Trials (RCTs), Controlled Trials and Multicentric studies, which evaluated the clinical differences between patients rehabilitated with dental implants who take these drugs or less, were included. This manuscript that brings together different clinical studies and evaluates different outcomes, aims to clarify this therapy in certain patients, providing a contribution where currently the literature appears conflicting.

## Materials and methods

### Eligibility criteria

The inclusion criteria are as follows:

- Recent studies (last 15 years), to avoid the risk of running into drugs that are no longer used and safe.
- Human studies
- Studies on subjects with no other absolute contraindications to implant therapy
- Studies concerning completed implant-prosthetic therapy
- Studies which are present and legible or accessible: title-abstract-full text.

### Information sources

The research sources are the most common and reliable scientific research channels according to the scientific community:

- Scopus Elsevier®
- Web of Science®
- Google Scholar®
- Pubmed®
- MDPI® database

### Search strategy Selection process

The search strategy planned to insert the following word string in the information sources, according to the previous paragraphs:

"(biphosphonate) AND (dental implant)".

Thanks to the filters of the sources of scientific information, it is possible to perform a quick screening of the results so as to reduce the work by the individual authors in case there are manuscripts not of interest with the topic. Some of the filters that were used included considering recent studies (15 years), human studies, clinical studies (RCTs, Multicentric, Clinical Trials). These filters have been applied to all used information sources engines.

Furthermore, recent works were considered, in accordance with inclusion and exclusion criteria, and only some types of studies were selected, using the filters already present in the scientific information sources.

Subsequently, once the studies were obtained, they were subjected to a first screening through independent reading by the authors of "title" and "abstract", and then the full text was read (logically, if they were not discarded earlier as they did not meet the inclusion criteria already when reading "title" or "abstract"). Authors independently analyzed results with this process: Title-Abstract-Full text reading.

The revision method is in accordance with the PRISMA statement, with methodology and subdivision in the manuscript according to their parameters, as well as with the presence of the PRISMA flow chart [12–14].

This review is registered at PROSPERO with ID number 342214 with date 25/06/2022. PROSPERO is an international database of prospectively registered systematic reviews in health and social care.

As regards the identification of the study objective, he also made use of the PICO—Population Intervention Comparison Outcome system [15], with the following question:

Does implant therapy have different outcomes in patients who undergo bisphosphonate drug therapy than in patients who do not take bisphosphonates?

and as additional questions:

What about clinical and radiographical outcomes of dental implant therapy (paragraph 2.4)?

### Data items

The outcomes considered are all those extrapolated from the individual articles as outcomes:

- Bleeding on Probing (BoP);
- Probing Depth (PD);
- Mobility of dental implant;
- Thread exposure (TE);
- Bone Marginal loss;
- implant stability quotient (ISQ);
- Bone mineral density (BMD);
- Implant survival;
- Soft tissue condition.

### Study risk of bias assessment

The risk of bias was assessed according to the studies and method proposed by Cochrane and Higgins et al. (RoB 2) [16–21].The risk of bias between the studies was assessed, in addition, as there were randomized clinical trials, the level of bias within the individual studies was assessed.

The analysis of the citations was carried out with the Fi-index Tool [22] on the first author using Scopus Elsevier® as a search engine. The pre and post index difference result is 0.35, low and tolerable given the inherence of the citations and the line of scientific research.

### Synthesis methods

The studies were read by the authors individually and were subsequently discussed and analyzed, each author created tables and cards with the main results of all the articles included, these were reviewed and are exposed in the "results" and "discussion" sections. The synthesis was done manually by the authors, any doubts were clarified by a third expert author (G.C.).

## Results

### Study selection

**Fig. 1:**
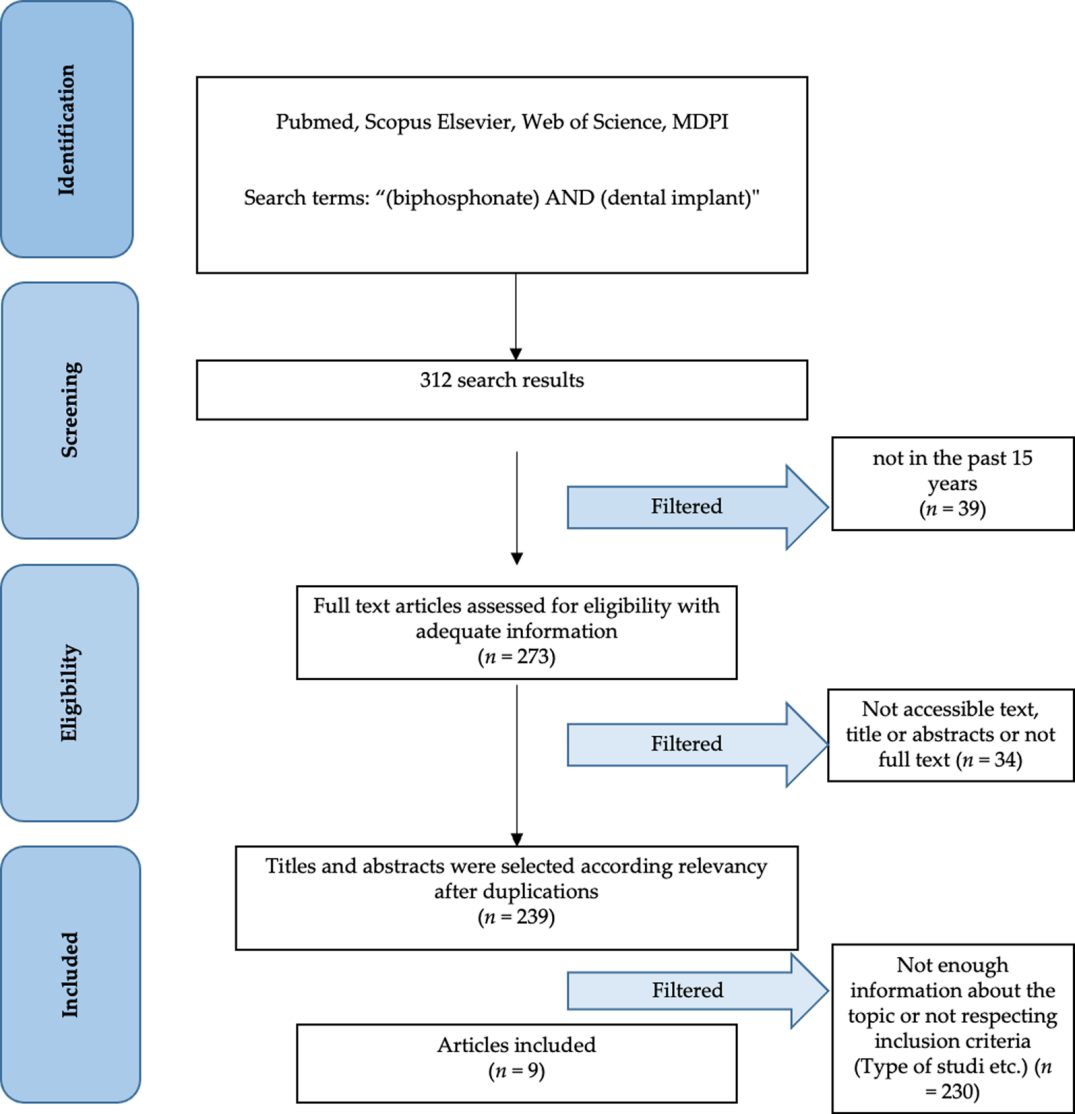
PRISMA flow chart

The studies taken into consideration are the result of a careful analysis by the authors. From a first survey on scientific search engines, as explained in paragraph 3.2, 312 works were obtained. In this case there are studies dating back to 1978. Only the Randomized Controlled Trial (RCT), Multicentric studies and Clinical Trial studies are taken into consideration, obtaining a number of 273 results. Following an analysis of the results, by first reading titles and abstracts, and in the event of uncertainty of the full text by two independently authors, the results selected and compatible with the review criteria are 9. According to the review method used (see Materials and Methods section), the PRISMA flow chart (Fig. 1) is presented. Some examples of the studies that were collected through the keywords by the use of scientific search engines, but that were excluded after an analysis by the authors are these: Aspenberg et al. [23] study, was about implant used for hip fracture and not about dental implant; Meidan et al. [24] paper was about the use of technetium 99 m-methylene diphosphonate scintigraphy for the clinical evaluation of peri-implant tissue, and not correlated to bisphosphonate therapy and new dental implants insertion; Trbakovic et al. [25] manuscript was about the use of granular calcium phosphate compound and a composite bisphosphonate-linked hyaluronic acid-calcium phosphate hydrogel in a rabbit model for bone regeneration.

### Study characteristics

The main characteristics of the selected studies are included in Table 1 below respecting the following parameters (Table 1):

- Authors—Authors of the single study
- Year—Year of publication of the study
- Type of study—Article type
- Control Group—Describe the presence of a control group and type
- Follow-up—follow up time duration

**Table 1:** Studies characteristics

Authors	Year	Type of study	Methodology	Control group	Follow up
Abtahi et al. [26]	2019	RCT	Double-blind, split-mouth	Uncoated zoledronate dental implant	8 weeks
Abtahi et al. [27]	2016	RCT	Double blind, split-mouth	Uncoated zoledronate dental implant	5 years
Tallarico et al. [28]	2016	Multicentric Study	–	–	3 years
Zuffetti et al. [29]	2015	RCT	Split-mouth	No topical administration of bisphosphonate	1 year
Mozzati et al. [8]	2015	Clinical Trial	–	–	10 years
López-Cedrún et al. [32]	2013	Multicentric Study	–	–	3 years
Griffiths [33]	2012	RCT	Split-mouth	No bisphosphonate oral administration	18 months
Abtahi et al. [34]	2012	RCT	Double-blind, split-mouth	Uncoated zoledronate dental implant	6 months
Shabestari et al. [35]	2010	Multicentric Study	–	–	5 years

### Results of individual studies

The results present in these selected studies will be listed below in Table 2 and divided as follows (Table 2):

- Authors—Authors of the single study
- Sample size—Sample size of the study
- Type of groups—subdivision of the groups and type
- Main outcomes results—principal data results of the study (separated by semicolon)
- Statistic—statistical data (respectively to “Main outcomes results" and separated by semicolon)

**Table 2:** Selected study individual results

Authors	Sample size	Type of groups	Main outcomes results	Statistic
Abtahi et al. [26]	32 dental implants on 16 patients	1. Zolendronate coated dental implant	0.17 mm of marginal bone loss between groups	P < 0.006
		2. Uncoated dental implant	No implant stability differences	
Abtahi et al. [27]	32 dental implants on 16 patients	1. Zolendronate coated dental implant	Marginal bone loss difference between groups at 18 month was 0.50 mm	P = 0.04
		2. Uncoated dental implant	Marginal bone loss difference between groups at 5 years was 0.34 mm	P = 0.04
Tallarico et al. [28]	98 dental implants in 32 patients	1. Dental implants in 6 month alendronated administration stopping patients	Dental implant success 98%; Prostheses success 98%; Survival rate 100%; Median marginal bone loss 1.35 mm; No differences between 1, 2 and 3 years of follow up on marginal bone loss Soft peri-implant tissue with positive parameters;	Differences on 1, 2 o 3 years of follow up P = 0.059
Zuffetti et al. [29]	155 dental impla ts in 39 patients	1. Standard dental implant procedure	Dental implant survival was 100% in test group and 91.3% in control group	P = 0.001
		2. Standard dental implant procedure + a 3% chlodronate and surfactant solution rinse both at the implant surface and implant site	Marginal bone loss of 0.85 mm in test group and 1.12 mm in control group after one year	P = 0.15
			Marginal bone loss of 0.98 mm in test group and 1.26 mm in control group after 5 years	P = 0.15
Mozzati et al. [8]	1267 dental implants on 235 patients	1. Oral bisphosphonate administred patients	Dental implant failure rate of 6.8%	–
López-Cedrún et al. [32]	57 dental implants on 9 patients	1. Oral bisphosphonate administred patients	–	–
Griffiths [33]	10 patients	1. Standard dental implant procedure	Bone mineral density (BMD) evaluation was lower in oral bisphosphonate patients	–
		2. Standard dental implant procedure with 70 mg tablet or placebo of alendronate weekly for 6 months		
Abtahi et al. [34]	32 dental implants on 16 patients	1. Pamidronate and ibandronate coated dental implant	Dental implant success rate of 100%; Larger implant stability quotient (ISQ) for bisphosphonate coated dental implant at 6 months	100%
		2. Uncoated dental implant	Bone marginal loss was higher in non-coated dental implants at 6 months	P = 0.0001; P = 0.003
Shabestari et al. [35]	46 dental implants in 21 patients	1. Dental implant surgery in patients that started oral bisphosphonate therapy after healing	Bleeding on Probing (BoP); Probing Depth (PD)	Not significant differences between groups in all outcomes (P < 0.05)
		2. Dental implant surgery in patient who undergone oral bisphosphonate therapy	Mobility; Thread exposure (TE)	

### Results of syntheses

In Abtahi et al. [26] study, zoledronate coated vs uncoated dental implants were compared. These two types of implants were indistinguishable by a blinded operator. A total of 30 dental implants in 16 patients were placed. Implant stability values were constant over follow-up time, with no statistical differences. About blind qualitative scoring, 13 of the 15 control implants and two of 15 coated implants showed small marginal bone defects. A second study of Abtahi et al. [27] evaluated the same patients with 5 years of follow-up. Marginal bone loss increased over time in both groups, but results were satisfactory for test group. For example, at 5 years the bisphosphonate coated dental implants performed a 0.20 mm of marginal bone loss vs 0.70 of median value for dental implants in control group. Unfortunately, 2 patients died at 5 years follow up, so there are 4 dental implants missing (Table 3).

**Table 3:**
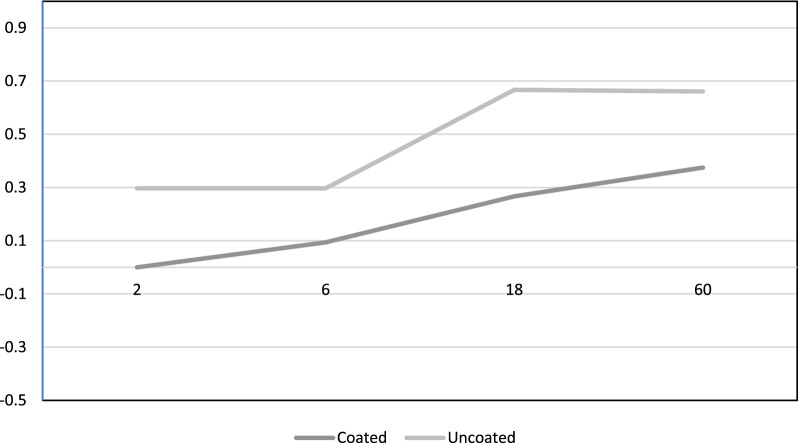
Abtahi et al. [26, 27] studies dental implants bone marginal loss between groups

X axis: bone marginal loss in mm; y axis: follow up time in months

Tallarico et al. [28] study evaluated the effect of dental implant surgery on bisphosphonate administered patients. After a pharmacological prophylaxis 98 dental implants were inserted (Table 4).

**Table 4:** Tallarico et al. [28] dental implant surgery prophylaxis in bisphosphonate patients

6 months before surgery	Suspension of the BPs before surgery and if possible after surgery Professional hygiene
7 days before and after surgery	Amoxicillin and clavulanic acid, 1 tablet every 12 h (2 g for day) Metronidazole 250 mg; 2 tablets every 8 h (1.5 g for day) Chlorhexidine gluconate 0.2% (oral rinse)
Day surgery	Oral rinse with chlorhexidine gluconate 0.2% for 1 min Flapless or mini-flap approach Copious irrigation during implants sites preparation Two-stage implants placement
Post-surgical	Ibuprofen 600 mg every 8 h for 2 days (later on if needed) Periodic (3–6 months) follow-up

Only one of 98 dental implants failed during the healing period in < 90 years old patients, and no other dental implants or prosthetic failures or complications occurred during follow-up period. 155 dental implants in 39 patients were placed in Zuffetti et al. [29] study. These patients were fully or partially edentulous. Prosthetic phase started 10 weeks after dental implant insertion, two groups were subdivided: test and control groups. Test group was characterized by a 3% chlodronate solution mixed with a surfactant (Tween-20) at a 1:3 ratio topically administered both at the implant surface and at the implant preparation bone site [30]. A total of 6 dental implant failed in control group vs zero in test group. Furthermore, results showed better results in test group than in control group regards: Implant survival, bone marginal loss and complications. Mozzati et al. [8] realized a very large sample size study about this topic. They evaluated more than 200 patients with oral bisphosphonate therapy and inserted more than 1000 dental implants after a standard antibiotic prophylaxis [31]. During 24 months follow-up, there were no BRONJ (Bisphosphonate related osteonecrosis of the jaw) cases. 16 dental implants failures were recorded on 16 different patients with absence of infection but represented by mobility. Authors, so highlighted patients risk factors related to a specific bisphosphonate therapy (risedronate) and other patients-related features as diabetes, corticosteroid therapy or smoking. Furthermore, regenerative surgery maneuvers or post-extractive dental implant surgery seems to represent a risk factor. López-Cedrún et al. [32] with their multicentric studies evaluated 9 patients with BRONJ associated to dental implants. Most of cases interested the posterior mandible with the presence of pain and swelling, with suppuration in other cases. The bone exposition was present, with radiolucent lesions at radiographical examination too. The treatments consisted of dental implant removal and bone sequestrectomy. Griffiths [33] in his pilot study, evaluated the differences in Bone Mass Density (BMD) around dental implants in patients who performed a therapy with oral bisphosphonates at time of dental implant surgery or after dental implant surgery. He performed CT scans and evaluated BMD with Hounsfield (HU) unit scale. A less evident decreasing trend in BMD surrounding an implant when alendronate was administered for 6 months after the implant had successfully undergone osseointegration for 6 months. Abtahi et al. [34] in their oldest study (2012), already evaluated the effect of bisphosphonate coated dental implants on bone tissue. In this case, dental implants were placed in a chamber, and then baked at 60 °C until 150 °C. This process took to have a cross-linked layer of fibrinogen with small amounts of pamidronate and ibandronate covalently bound to the titanium surface. They did not lose dental implants during follow-up time and there were no surgical complications. According to Authors, the bisphosphonate coated dental implants showed a larger increase in ISQ value, as showed in Table 2. Marginal bone loss, showed better results in bisphosphonate coated implants vs uncoated ones (Table 5). Shabestari et al. [35] in their multicentric study, evaluated the effect and some survival parameters of dental implant surgery in two different groups of patients (Table 2). They did not show any differences on BoP, PD or TE between the group that received dental implants during bisphosphonate therapy and the group who became bisphosphonate therapy after healing. No one of the implants showed mobility and all patients were considered peri-implantitis free. According to Shabestari et al. [35], implant location, the presence of opposing dentition, or prosthetic rehabilitation type had no influence on clinical and radiological parameters after dental implant surgery in BP patients. Authors followed-up a disomogeneous group of patients for 5 years in some cases after implant insertion.

**Table 5:**
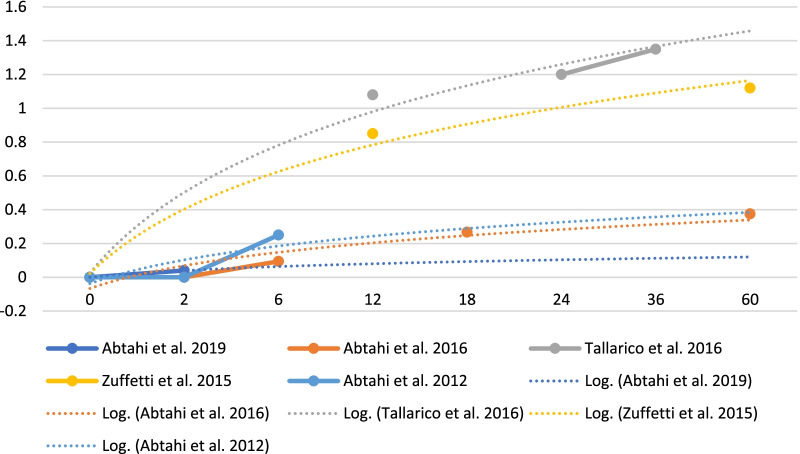
Bone level changes on dental implants affected by bisphosphonates

X axis: marginal bone levels variations in mm; Y axis: time in months. Data sources have been specified

### Reporting biases

The bias of the studies included in the review was assessed according to what is described in the materials and methods section.

Below, Table 6 further clarify the results of the risk of bias present, that could be defined as low.

**Table 6:** Risk of bias definition

	Abtahi et al. [26]	Abtahi et al. [27]	Tallarico et al. [28]	Zuffetti et al. [29]	Mozzati et al. [8]	López-Cedrún et al. [32]	Griffiths [33]	Abtahi et al. [34]	Shabestari et al. [35]
Random Sequence generation	+	+	−	+	−	−	+	+	−
Allocation concealment	+	+	+	+	+	+	+	+	+
Blinding of participants and personnel	+	+	−	−	−	−	−	+	−
Blinding of outcome data	+	+	−	−	−	−	−	−	−
Selective reporting	+	+	+	+	+	+	+	+	+
Other bias	+	+	+	+	+	+	−	+	+

### Certainty of evidence

Table 5 reports important data regarding changes in marginal bone levels around implants "associated" with bisphosphonate drugs. Somehow it is possible to easily identify a trend from this chart. Certainly, the marginal bone loss around dental implants, just like teeth, is constant and progressive, and is defined by the recent consensus conferences as approximately 0.1 mm per year [36]. However, already from Table 3 it is possible to highlight differences between implants coated with bisphosphonates and uncoated implants. This suggests that in some way the effect of these molecules contrasts bone resorption, even in this clinical condition. In Table 5 all the studies that took into consideration the marginal bone has been included, thus obtaining a homogeneous graph of results, which compared over time, show a trend and overlapping values. Logarithmic trend lines were also drawn, which somehow follow the results obtained by the individual authors, but show what could be the progression over time. Furthermore, in Table 7 below, those trend lines up to 15 years have been projected to understand how limited bone loss could be on implants, associated in some way with therapy with these drugs.

**Table 7:**
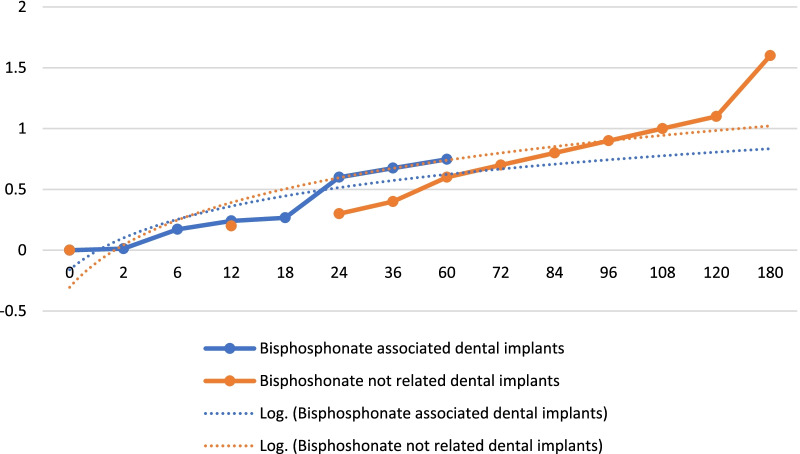
Trend lines regarding bone marginal loss on implants "associated" with bisphosphonates and non "associated" implants

X axis: median bone marginal loss, Y axis: time in months

In this case (Table 6), the mean values of all studies with implants associated with bisphosphonate drugs were considered, admitting the missing values as 0. In the graph about dental implants not related to bisphosphonates, on the other hand, the parameter of 0.1 mm per year of bone loss was set. It is necessary to clarify that by "associated" with bisphosphonates it means either a patient undergoing drug therapy or an implant coated with these molecules. The data after 10 and 15 years actually, in the logarithmic trend line, show superimposable results. By carrying out a paired t-test with two samples, and inserting the values, in fact has been noticed that the result is not statistically significant. The two-tailed P value equals 0.1483. By conventional criteria, this difference is considered to be not statistically significant.

## Discussion

According to Abtahi et al. [26] there are no statistical differences between the use of bisphosphonate coated dental implant or uncoated ones during the early healing phase. Uncoated dental implants just presented more marginal bone loss than test group. Another study of Abtahi et al. [27] with 5 years of follow up, concludes that bisphosphonate coated dental implant could prolong preservation of the peri-implant marginal bone. Tallarico et al. [28] reported that bisphosphonate therapy in patients did not significantly affect implant survival and prosthetic rehabilitation success rate. Authors specify that an accurate treatment time selection and a minimally invasive surgical approach are required. Zuffetti et al. [29] showed how topical administration of bisphosphonate drugs could positively affect dental implants survival and pre and post loading phases. Mozzati et al. [8] reported no cases of BRONJ after dental implant surgery in oral bisphosphonate treated patients, these procedures so, could be safely done but attention to techniques that enhance and support healing as platelet concentrates. López-Cedrún et al. [32] in their study, showed how dental implant associated BRONJ have similar clinical features and outcomes of treatments of those seen in patients with BRONJ with no dental implants. Lesions may develop in early or late phase of dental implant or prosthetic rehabilitation. Griffith [33] concluded that bisphosphonate could suppress regional phenomenon related to healing if patients take these drugs before and at time of implant surgery, but these drugs could have positive effects after 6 months under normal conditions. Abtahi et al. [34] already in 2012, suggested that a thin bisphosphonate fibrinogen coating, could improve the osteointegration of dental implants in the human bone. Surely, according to authors, this could open new possibilities in orthopedic surgery, through osteoporotic bone and dental rehabilitation. Still many issues about bisphosphonate-associated osteonecrosis remain unclear, according to Shabestari et al. [35], this multicentric study did not show correlation between this pathology and dental implant surgery. However, the study had a big limitation, represented by a limited pool of patients and furthermore not all patients completed the same follow-up time.

Bisphosphonates are stable molecules analogous to inorganic pyrophosphates and have been shown to be effective in the treatment of osteolytic lesions associated with bone metastases, multiple myeloma, malignant hypercalcemia, Paget's disease and osteoporosis. Several publications in recent years have suggested that osteonecrosis of the jaw is associated with bisphosphonate therapy. Strategies for diagnosing and managing patients with bisphosphonate-induced osteonecrosis of the jaw are very difficult. It is important for patients to be informed of the risk of this complication, so that they have the opportunity to assess the need for dental treatment before starting therapy [37]. If osteonecrosis of the jaw is present, management should be conservative: oral chlorhexidine and antibiotics. Surgical treatment should be reserved for those patients who are symptomatic. Preventive therapeutic measures must be taken before, during and after bisphosphonate treatment, as stated in the guidelines. Currently, it is a well-known fact that this type of BP molecules is in some way connected to bone necrosis, which is mostly seen in the jaws. This is a rare complication but, obviously, of considerable severity and difficult to treat. Bisphosphonates have a high affinity for bone tissue, in particular they act by inhibiting the activity of osteoclasts. The result will obviously be the reduction of bone matrix resorption. Although osteonecrosis has so far been reported mainly in patients undergoing intravenous administration of bisphosphonates, an increasing number of cases are reported among patients undergoing oral bisphosphonates for the treatment of osteoporosis or Paget's disease. The first reported cases of osteonecrosis of the jaws associated with bisphosphonate therapy date back to 2003 [11]. Osteonecrosis of the jaw or jaw is a disabling disease of a progressive nature and with little tendency to healing. The onset signs are very subtle and extremely variable and range from gum inflammation that does not heal, to the loss of a tooth, to the slow or non-healing of an extraction, to a periodontitis picture, to the presence of dental abscesses or fistulas in the mouth or externally, on the skin. The dental approach is fundamentally based on prevention and certainly on the direct relationship with the other specialists involved in patient management. In the event that some oral surgery interventions are necessary and cannot be postponed, the dentist decides under close advice and collaboration with the specialist doctor how to proceed for the treatment of infection, pain, in order to reduce/avoid the risk of osteonecrosis. adopting specific treatment protocols and using fewer traumatizing techniques for the tissues. Any dental extraction or surgical procedure should be completed before the start of bisphosphonate therapy, taking into account the time required for healing. The risk/benefit profile should be considered for each patient before starting chronic therapy and in cases where there are local or systemic risk factors for osteonecrosis of the jaw, the possibility of alternative estrogen therapy should be considered, such as in patients with postmenopausal osteoporosis. Since the primary objective is the elimination of all potential sites of infection, patients should be informed on the best way to treat oral hygiene. In addition, regular dental visits should be scheduled [38–42].

Given that osteonecrosis of the jaw is more frequently associated with traumatic dental procedures for the bone, endodontic therapies should be preferred to dental extractions and invasive periodontal procedures in predisposed patients. Dental implants should also be avoided. As a result, literature shows that a careful examination of the patients under or previous bisphosphonate therapy before planning of dental insertions is very important for successful results. Even low, there is always a risk of developing bisphosphonate-induced osteonecrosis of the jaw, as well as the risk failure of implants [43]. Additionally, some studies show that some types of bisphosphonate molecules may be more related to the risk of BRONJ if the patient receives a dental implant. For example, one molecule that is reported as more risky is risedronate. Other factors related to a higher risk of BRONJ in case of implant surgery are smoking, diabetes and the use of corticosteroids [8]. Before undertaking any surgical-implant therapy, it is necessary to acquire and analyze a complete medical history of the patient and in the case of the presence of bisphosphonate therapy, the duration of treatment must be confirmed, as well as taking into consideration the route of administration of the itself.

### Limitations

The main limitation of this study is that the result represented was not retrieved directly from analysis of clinical cases, but the data was extrapolated from different studies which showed a scarce diversity of protocols among them, such as types of surgeries, year of publication, and type of drug taken by the patient. Unfortunately, even the outcomes of the individual studies are not easily superimposed and it was not possible to conduct a meta-analysis. All the single outcomes have been reported in the Table 2 with the corresponding statistical result. Only the overlapping outcomes were subjected to further statistical analysis. Despite the use of different sources of scientific information, often some manuscripts can be excluded from the filters of electronic search engines. It is also always important to bear in mind that some manuscripts may present different keywords, incorrect or not in accordance with the Medical Subject Headings (Mesh) word.

## Conclusions

Thanks to this study it is possible to clarify the effects of these molecules on the bone, during and after the implant-prosthetic rehabilitation therapy.

Table 5 reports important data regarding changes in marginal bone levels around implants "associated" with bisphosphonate drugs. Somehow it is possible to easily identify a trend from this chart. Certainly, the marginal bone loss around dental implants, just like teeth, is constant and progressive, and is defined by the recent consensus conferences as approximately 0.1 mm per year [36]. However, already from Table 3 it is possible to highlight differences between implants coated with bisphosphonates and uncoated implants. This suggests that in some way the effect of these molecules contrasts bone resorption, even in this clinical condition. In Table 5 all the studies that took into consideration the marginal bone has been included, thus obtaining a homogeneous graph of results, which compared over time, show a trend and overlapping values. Logarithmic trend lines were also drawn, which somehow follow the results obtained by the individual authors, but show what could be the progression over time. Furthermore, in Table 7 below, those trend lines up to 15 years have been projected to understand how limited bone loss could be on implants, associated in some way with therapy with these drugs.

In this case (Table 6), the mean values of all studies with implants associated with bisphosphonate drugs were considered, admitting the missing values as 0. In the graph about dental implants not related to bisphosphonates, on the other hand, the parameter of 0.1 mm per year of bone loss was set. It is necessary to clarify that by "associated" with bisphosphonates it means either a patient undergoing drug therapy or an implant coated with these molecules. The data after 10 and 15 years actually, in the logarithmic trend line, show superimposable results. By carrying out a paired t-test with two samples, and inserting the values, in fact has been noticed that the result is not statistically significant. The two-tailed P value equals 0.1483. By conventional criteria, this difference is considered to be not statistically significant.

The data extrapolated by this investigation offer an overview about guidelines to be followed in case of performing oral surgical treatment of bisphosphonates drug patients related. Implant surgery, if correlated with a suitable pharmacological prophylaxis, appears to be safe, for patients and for the predictability of our rehabilitations, the data show no statistically significant differences regarding the marginal bone loss around the implants, despite the values being slightly in favor. implants associated with bisphosphonates. Certainly, more randomized clinical trials, more multicentric studies, are needed to shed light on this condition that often limits the quality of life related to oral health (ORQoL) of our patients, and places the clinician in front of a dilemma and doubts that are still unresolved. full clarified by the guidelines.

Thanks to this study it is possible to clarify the effects of these molecules on the bone, during and after the implant-prosthetic rehabilitation therapy. Implant surgery, if correlated with a suitable pharmacological prophylaxis, appears to be safe, for patients and for the predictability of our rehabilitations, the data show no statistically significant differences regarding the marginal bone loss around the implants, despite the values ​​being slightly in favor. implants associated with bisphosphonates. Certainly, more randomized clinical trials, more multicentric studies, are needed to shed light on this condition that often limits the quality of life related to oral health (ORQoL) of our patients, and places the clinician in front of a dilemma and doubts that are still unresolved. full clarified by the guidelines.

## Data Availability

The datasets generated during the current study are not publicly available due to individual research work but are available from the corresponding author on reasonable request.
